# The transformations of cellulose after concentrated sulfuric acid treatment and its impact on the enzymatic saccharification

**DOI:** 10.1186/s13068-023-02293-4

**Published:** 2023-03-04

**Authors:** Shengbo Wu, Suan Shi, Ruotong Liu, Chun Wang, Jing Li, Lujia Han

**Affiliations:** 1 Engineering Laboratory for Agro Biomass Recycling & Valorizing, College of Engineering, China Agricultural University, Beijing, 100083 China; 2 School of Ecology and Environment, Beijing Technology and Business University, Beijing, 100048 China

**Keywords:** Cellulose, Sulfuric acid treatment, Crystalline structure, Transformation, Enzymatic saccharification

## Abstract

**Background:**

The dense structure of cellulose lowers its reactivity and hinders its applications. Concentrated sulfuric acid is an ideal solvent to dissolve cellulose and thus has been used widely to treat cellulose. However, the changes of cellulose after reaction with concentrated sulfuric acid at near-limit S/L ratio and its effect on enzymatic saccharification still need further investigation.

**Results:**

In this study, the interactions between cellulose (Avicel) and 72% sulfuric acid at very low acid loading conditions of 1:2 to 1:3 (S/L ratio) were studied for the enhanced production of glucose. The Avicel gradually transformed from cellulose I structure to cellulose II structure during the sulfuric acid treatment. Other physicochemical characteristics of Avicel also changed dramatically, such as the degree of polymerization, particle size, crystallinity index, and surface morphology. After acid treatment, both the yield and productivity of glucose from cellulose increased significantly under a very low enzyme loading of 5 FPU/g-cellulose. The glucose yields for raw cellulose and acid-treated (30 min) were 57% and 85%, respectively.

**Conclusion:**

Low loadings of concentrated sulfuric acid were proven to be effective to break the recalcitrance of cellulose for enzymatic saccharification. A positive correlation between cellulose CrI and glucose yield was found for concentrated sulfuric acid-treated cellulose, which was opposite to previous reports. Cellulose II content was found to be an important factor that affects the conversion of cellulose to glucose.

**Supplementary Information:**

The online version contains supplementary material available at 10.1186/s13068-023-02293-4.

## Introduction

As one of the main components of plants, cellulose is an almost inexhaustible material. The proper use of cellulose could provide solutions to environmental and energy problems. Cellulose can be used in the replacement of petroleum to produce various products, such as biofuels [1, 2], platform chemicals [3], and advanced materials [4], among others. One big advantage of cellulose is its carbon neutrality, especially in the case of biofuels [5, 6]. The carbon in biofuels is from the carbon absorbed by plants from the atmosphere, so the burning of biofuels will not burden the carbon cycle [7]. Research has shown that cellulose has four different crystal forms: I, II, III, and IV [8]. Natural cellulose has a type I cellulose structure, which is widely found in plant cell walls and is commonly used in the apparel, cosmetic, and pharmaceutical industries [9]. Cellulose II can be made by dissolution and regeneration or alkaline mercerization of natural cellulose, and it is the most thermodynamically stable form of cellulose due to the presence of additional hydrogen bonds [10]. The crystallinity of cellulose II is lower than natural cellulose. Cellulose II is used in a wide range of industrial applications, such as the production of smart materials, packaging materials, biomedicine, reinforcement materials, and biofuels [9]. Cellulose III can be prepared by liquid ammonia treatment of cellulose I or cellulose II, which is a precursor to many cellulose derivatives because of its highly reactive and unstable nature [11, 12]. Cellulose IV is used to be called “high-temperature cellulose” because its production requires heating at high temperatures over 250 °C [13]. Cellulose IV can only be transformed from cellulose II or III and it cannot be made directly from cellulose I. Due to its preparation at high temperatures, cellulose IV has better stability than cellulose III and it is often used in food processing [14]. Different variants of cellulose can be distinguished by X-ray diffraction (XRD), Fourier-transform infrared spectroscopy (FTIR), CP/MAS ^13^C-NMR, etc. [15]. All crystal forms of cellulose consist of D-glucose linked by β-1,4 glycosidic bonds. Microfibers are polymerized by hydrogen bonds and van der Waals forces, the large number of internal and intermolecular hydrogen bonds in cellulose gives it a dense structure, which greatly reduces the reactivity of cellulose and requires pretreatments before it can be used effectively. Pretreatment methods for cellulose include physical, chemical, biological, and combined treatment methods. The acid treatment method is widely used due to its low cost, easy operation, and diversified product performance. Sulfuric acid can effectively penetrate the cellulose structure at high concentrations, disrupting the orderly stacking of molecular chains and breaking the hydrogen bonds within the cellulose [16, 17]. Sulfuric acid of 72% concentration is commonly used for cellulose treatment since it can readily dissolve cellulose at room temperature [18, 19]. It can be used to produce non-crystalline cellulose [20], nanocrystalline particles (NCP) [21], and also to measure cellulose content in lignocellulosic biomass [22].

Plenty of studies have been carried out to increase the glucose yield from enzymatic hydrolysis of pure cellulose or lignocellulose. Yeh et al. used a media mill to reduce the average particle size of microcrystalline cotton fibers to the submicron level and obtained a high glucose yield of 60% with 10 h of hydrolysis [23]. Wiman et al. found a significant correlation between enzymatic hydrolysis rate and BET area when treating spruce slices using the steam pretreatment method [24]. The degree of polymerization (DP) is also an important character of cellulosic materials in terms of functional modeling of enzymatic cellulose hydrolysis [25]. The crystallinity index (CrI), as an indicator of the degree of crystallization, is also one of the most important indicators of the reactivity of cellulose. Jiang et al. used combined pretreatment of corn stover with deacetylation and liquid hot water and Peng et al. combined pretreatment of cellulose with BM and microwave irradiation had the same finding: the enzymatic hydrolysis rate was much more sensitive to crystallinity than BET area and DP [26, 27].

Previous studies normally used a large amount of acid to dissolve cellulose, which would bring in a high cost of waste acid treatment. Also, there are inconsistent conclusions about which factor glucan digestibly is most sensitive to. In this study, the interaction between concentrated sulfuric acid and cellulose under high solid conditions was studied, and the correlation between physiochemical factors and enzymatic hydrolysis was systematically analyzed. The enzymatic hydrolysis performance of cellulose samples with the same crystallinity obtained by different treatments was also compared to analyze the influence of cellulose crystallinity and crystal form.

## Results and discussion

### Effect of sulfuric acid treatment on the physical structure of cellulose

**Fig. 1:**
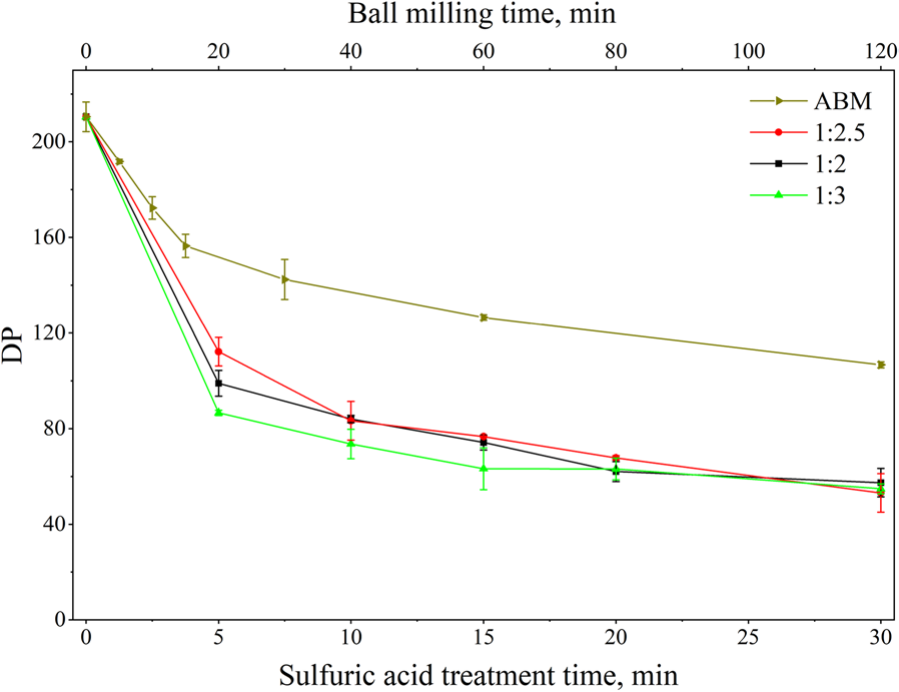
The DP of cellulose samples after sulfuric acid treatment and BM

Avicel PH101 is exemplary crystalline cellulose with a high DP of ~ 200 and a relatively small particle size of ~ 50 µm. The sulfuric acid treatment of Avicel made significant changes in its DP, particle size distribution, and also surface morphology. A rapid reduction of DP from 210 to around 100 was observed in the early stage (within 5 min) of sulfuric acid treatment on Avicel for all three acid-loading conditions (Fig. 1). As the acid treatment continuous, the rate of DP reduction became much slower and the DP stabilized at ~ 50 with 30 min of acid treatment. The cellulose-to-acid ratio did not show a clear impact on the DP changes as shown in Fig. 1. In comparison, the BM treatment was not as effective as the sulfuric acid treatment on the DP reduction of cellulose. After 120 min of BM treatment, the DP of the Avicel sample was lowered to 107, which was higher than the sample treated with sulfuric acid for 5 min. It has been demonstrated that cellulose samples with lower DP will have higher enzymatic yields [28, 29].

**Fig. 2:**
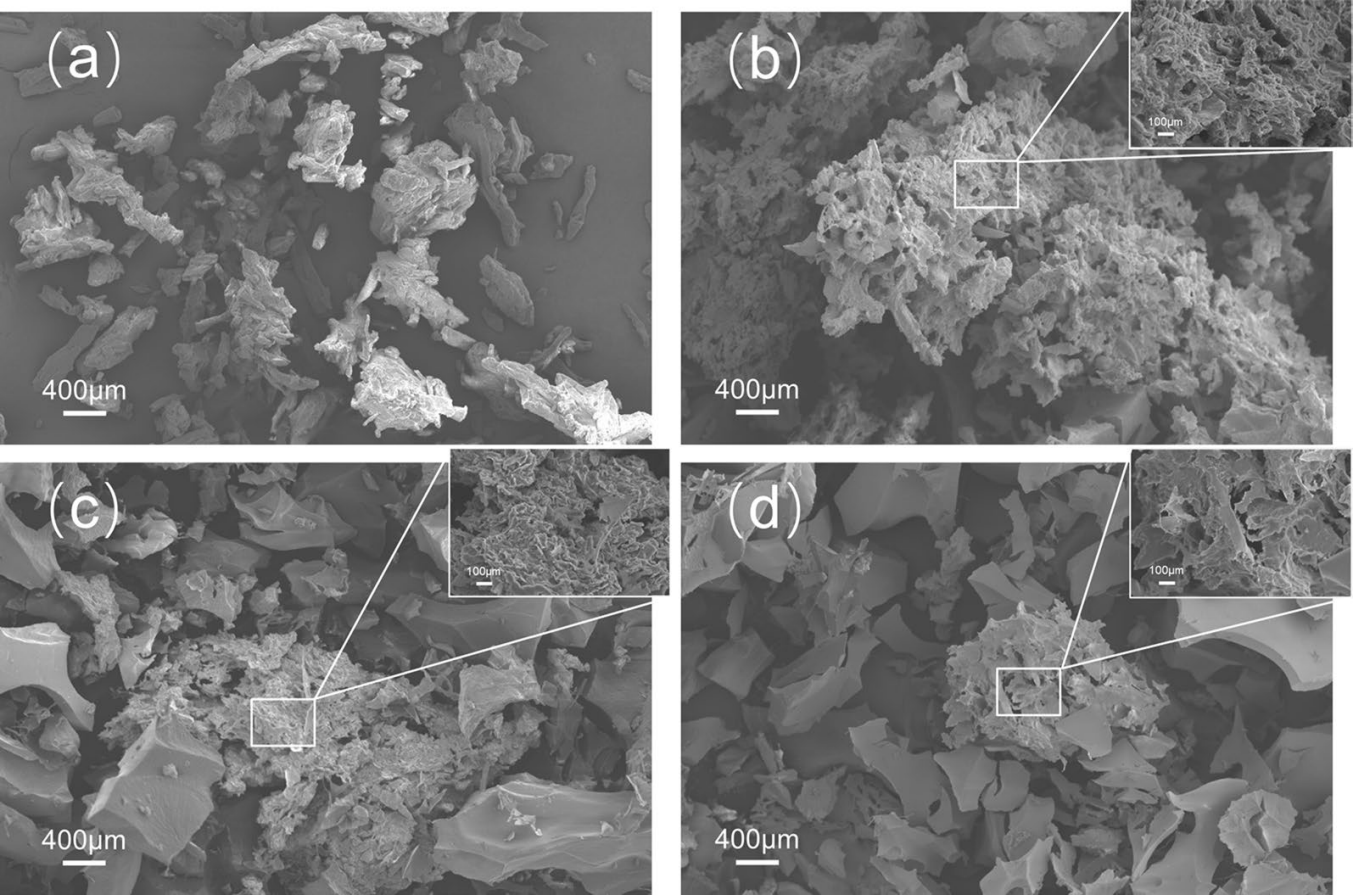
SEM images of cellulose samples **a** Avicel PH101, **b** 1:2.5–5 min, **c** 1:2.5–15 min, **d** 1:2.5–30 min

Figure 2 shows the SEM images of cellulose samples treated under 1:2.5 acid condition, the rest two cellulose-to-acid loadings had similar results and the SEM images can be found in the supplementary material (Additional file 1: Fig. S1). It can be observed that the cellulose particle became significantly larger after 5 min sulfuric acid treatment due to the particle aggregation during the process. The formation of larger cellulose particles would lower the surface-to-volume ratio. On the other hand, the cellulose surface after treatment became porous compared to raw Avicel, which would increase the surface-to-volume ratio. These two opposite effects resulted in inconsistent surface area data, e.g., the surface area of Avicel after 1:2.5–5 min treatment increased from 0.850 to 1.191 m^2^/g, but the surface area of the sample from 1:2 to 5 min treatment was 0.709 m^2^/g. When Avicel was treated for a longer time, the particle size began to decrease as shown in Fig. 2. This might be due to the further reaction between concentrated acid and cellulose which broke the hydrogen bonds inside the cellulose polymer and made the coagulated granules gradually disrupt into smaller particles. It was also found that the morphology of treated cellulose changed from irregular shape to lamellar structure as shown in Fig. 2d, and the porosity decreased as a result (data not shown). A similar result was observed by Liu et al. when the cellulose was treated with carbon dioxide [30].

The particle size of the cellulose samples was measured and was expressed as median particle size (D_50_). As we can see from Table 1, the D_50_ data confirmed our conclusion from SEM images: the cellulose particle became larger after sulfuric acid treatments and the particle size decreased as the treatment went further. The D_50_ of 5 min samples from all three acid-loading conditions were all over three times of raw Avicel; when extending the treatment time to 30 min, the particle size reduced to ~ 100 µm, which was still greater than raw Avicel. Although discernable changes in the particle size were observed in sulfuric acid treatment, the particle size was still at the microns level and the numbers are in the same order of magnitudes. These changes in particle size may not impact the following enzymatic hydrolysis significantly. Ioelovich and Morag found that the particle size of cellulose had little effect on the conversion yield of cellulose to glucose [28], and Ji et al. found that the accessibility of cellulose increased substantially only when the particle size of cellulose reached the cellular scale [31].

**Table 1:** The D_50_ of cellulose samples after sulfuric acid treatment

	Avicel PH101	1:2		1:2.5		1:3	
		5 min	30 min	5 min	30 min	5 min	30 min
D_50_ (µm)	51.36 ± 3.71	186.6 ± 13.65	120.0 ± 7.65	170.2 ± 19.54	102.2 ± 7.07	237.40 ± 34.85	96.6 ± 7.15

### Effect of sulfuric acid treatment on the cellulose II content of Avicel

**Fig. 3:**
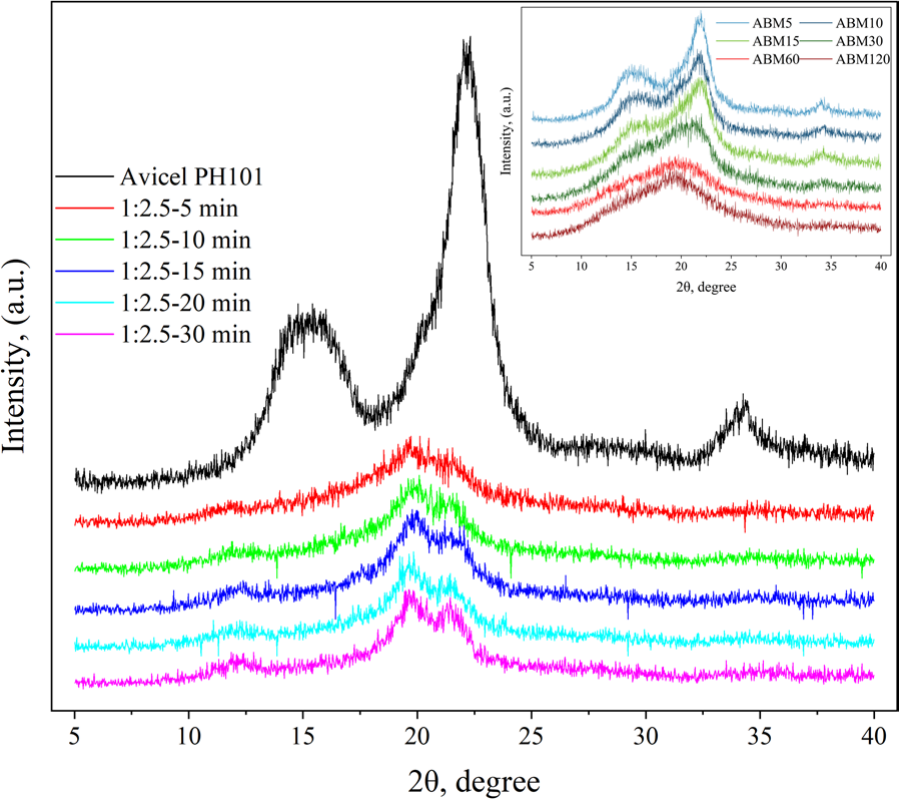
X-ray diffractograms of sulfuric acid-treated cellulose samples (cellulose-to-acid ratio of 1:2.5, the inset is X-ray diffractograms of ball-milled cellulose samples)

The cellulose samples before and after sulfuric acid and BM treatments were subjected to XRD analysis and the results are shown in Fig. 3. The raw Avicel sample showed diffraction angles of 14.6, 16.3, 22.4, and 34.3 degrees, representing the diffraction of the crystal plane of 101,10$\overline{1 }$, 002, and 040, respectively. These peaks are known to be the characteristic angle of cellulose I [32, 33]. When Avicel was ball milled for a series of durations, the sharp peaks gradually broadened (Fig. 3 inset), which indicates the loss of crystalline structure during the process [34, 35]. Although the crystallinity of the cellulose samples decreased during ball-milling treatment, these samples retained the cellulose I structure. When Avicel was treated with 72% sulfuric acid, the characteristic peak of cellulose I at 22.4° disappeared and two broad new peaks at 19.8° and 21.5° appeared, which are the characteristic peaks for cellulose II [36]. After sulfuric acid treatment, a new small peak also came out at a diffraction angle of about 12.1°. With increased sulfuric acid treatment time, these new peaks became more and more obvious in the XRD pattern of the samples, indicating a transition from cellulose I to cellulose II occurred when the severity of treatment increased. Similarly, Wei and Banker found that cellulose I was converted to cellulose II when treating cotton lint with 85% phosphoric acid [37]. A similar phenomenon also occurred when Zhang et al. treated microcrystalline cellulose with phosphoric acid [38]. Hashaikeh and Abushammala found the appearance of cellulose II structure in Avicel PH101 treated with sulfuric acid, attributed to the occurrence of recrystallization in the process of cellulose contact with organic solvent [39]. It is known that cellulose crystals are composed of cellulose chain layers bonded to each other by hydrogen bonds, and concentrated sulfuric acid could enter the cellulose chain layers to break the hydrogen bonds. When acid-treated cellulose was regenerated in water, the cellulose recrystallizes to produce cellulose II.

**Fig. 4:**
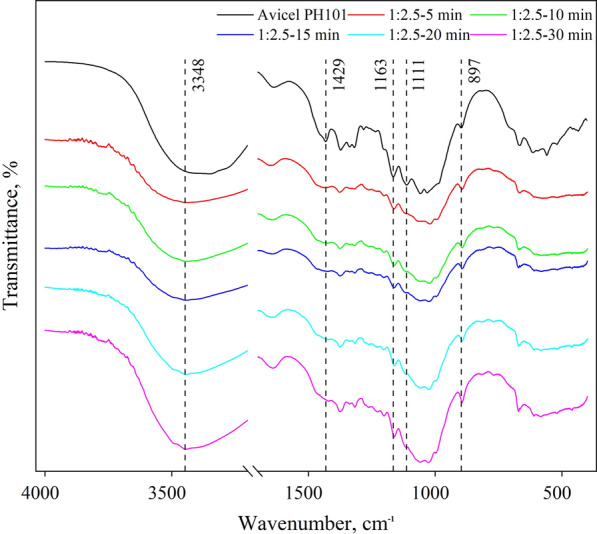
FTIR spectra of sulfuric acid-treated cellulose samples (cellulose-to-acid ratio of 1:2.5)

As shown in Fig. 4, the FTIR spectra of cellulose samples treated with sulfuric acid confirm the crystalline structure change. The peak at 897 cm^−1^ represented the β-glycosidic bond between the monosaccharides of cellulose I [40–42]. This peak was observed to shift to 893 cm^−1^ for sulfuric acid-treated samples, indicating the formation of cellulose II structure [43]. The peak at 1163 cm^−1^ is a strong characteristic peak in cellulose I which indicates the asymmetric stretching vibration of the C–O–C bond, and it was observed that in the samples treated with sulfuric acid, this peak moved to 1156 cm^−1^, which is the characteristic peak for cellulose II, indicating the formation of cellulose II [43]. Crystalline cellulose normally has spectrum peaks at 1429 cm^−1^ and 1111 cm^−1^ which are the stretching vibration of the CH_2_ bond and C–O bond, respectively [44]. But in cellulose II and amorphous cellulose, these two peaks are very weak and sometimes have slight shifts [43]. The weakened peaks at 1429 cm^−1^ and 1111 cm^−1^ for acid-treated samples suggested that the intramolecular hydrogen bond is broken and the CH_2_–OH undergoes a *tg* to *gt* conformational transition and a new intramolecular hydrogen bond is formed [45]. The decrease of these two peaks proved the change in crystallinity and crystalline structure during the acid treatment of cellulose. The absorption peak at 3348 cm^−1^ in the sulfuric acid-treated samples, which became larger with increasing treatment time, was attributed to the hydrogen bonding arrangement in cellulose II. It also demonstrated the crystalline conversion of cellulose I to cellulose II during the process of sulfuric acid treatment [9, 46].

The content of cellulose II (%) in cellulose samples can be calculated from their XRD profiles and the results are shown in Table 2. A short treatment (5 min) of Avicel with sulfuric acid dramatically increased the cellulose II content from near zero to over 70%, and the number kept increasing with increased treatment time. When the treatment time reached 30 min, the cellulose II content in the samples under all acid-loading conditions was close to 100%.

**Table 2:** The content of cellulose II in cellulose samples with sulfuric acid treatment

Cellulose-to-acid ratio	Content of cellulose II, %				
	5 min	10 min	15 min	20 min	30 min
1:2	70.44 ± 1.46	79.07 ± 1.56	90.00 ± 2.41	93.65 ± 2.90	97.52 ± 0.84
1:2.5	70.67 ± 2.06	79.14 ± 2.53	96.12 ± 1.57	96.55 ± 2.03	99.19 ± 0.21
1:3	70.30 ± 1.72	80.00 ± 2.49	99.15 ± 0.53	100.00 ± 0.00	100.00 ± 0.00

### Effect of sulfuric acid treatment on the crystallinity index of Avicel

**Fig. 5:**
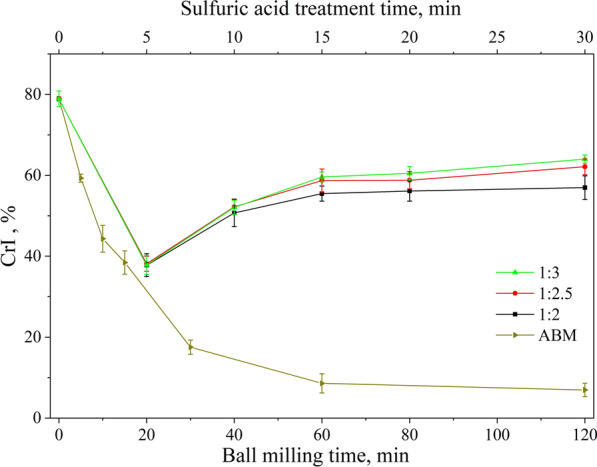
Crystallinity calculated from XRD of cellulose samples after sulfuric acid or BM treatment

The CrI of the raw and treated cellulose samples could be calculated based on the XRD profiles using Segal's formula for cellulose I-type samples and Azubuike’s formula for cellulose II-type samples. The CrI of raw Avicel was measured to be 78.90% and it quickly decreased to 38.43% with 15 min of BM treatment. The CrI of 60 min and 120 min BM treated samples were as low as 8.59% and 6.96%, respectively. Further increment of BM time did not give more decrystallization. This is because the dense structure of crystalline cellulose was destroyed after a long period of BM treatment to form large amorphous regions, leaving behind some stubborn crystalline regions which prevented the CrI go further down [47]. For the sulfuric acid-treated samples, the CrI decreased even more quickly to ~ 38% within 5 min of treatment for all three acid-loading conditions. With increasing sulfuric acid treatment time, the CrI increased. We do not have a clear explanation for this CrI increase now. One possible reason is that the longer the Avicel reacted in acid, the more sulfuric acid penetrated inside the cellulose chains. This led to more severe damage within the cellulose, which in turn brought in more recrystallization during the cellulose regeneration step in water after acid treatment. As we can see from Fig. 5, higher acid loading gave a higher CrI increase, this might be because there was more acid per gram of cellulose to achieve more cellulose internal disruption and thus increased CrI.

**Fig. 6:**
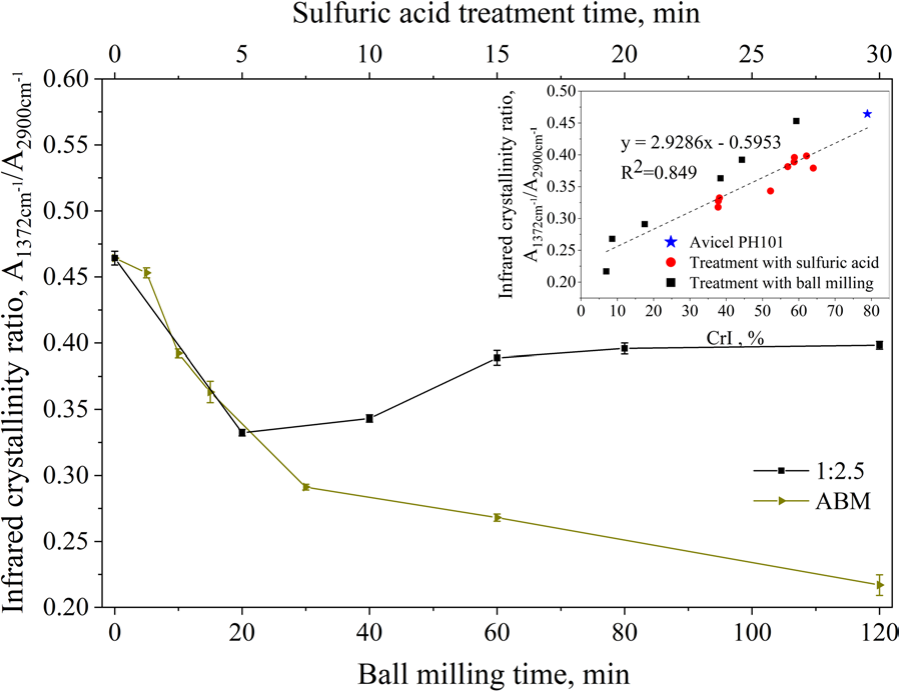
Infrared crystallinity ratio of cellulose samples after sulfuric acid or BM treatment (the inset depicts the relations between infrared crystallinity ratio and crystallinity from XRD)

The crystallization level of cellulose can also be measured by using the intensity ratio of 1372 to 2900 bands [43]. The results of the infrared crystallinity ratio calculated according to O'Connor's method are shown in Fig. 6. The changing pattern of the infrared crystallinity ratio under different conditions was similar to that calculated from XRD, and there was some deviation between the two methods. This is because these two calculation methods do not have a completely linear relationship. Figure 6 (inset) describes the regression dispersion of the ratio of crystallinity to infrared crystallinity with a correlation coefficient of 0.849, which was consistent with that described in the literature [43]. The results of FTIR spectroscopy analysis were consistent with XRD, which corroborated the effect of BM and sulfuric acid treatment on Avicel.

### Effect of sulfuric acid treatment on the enzymatic hydrolysis of cellulose

**Fig. 7:**
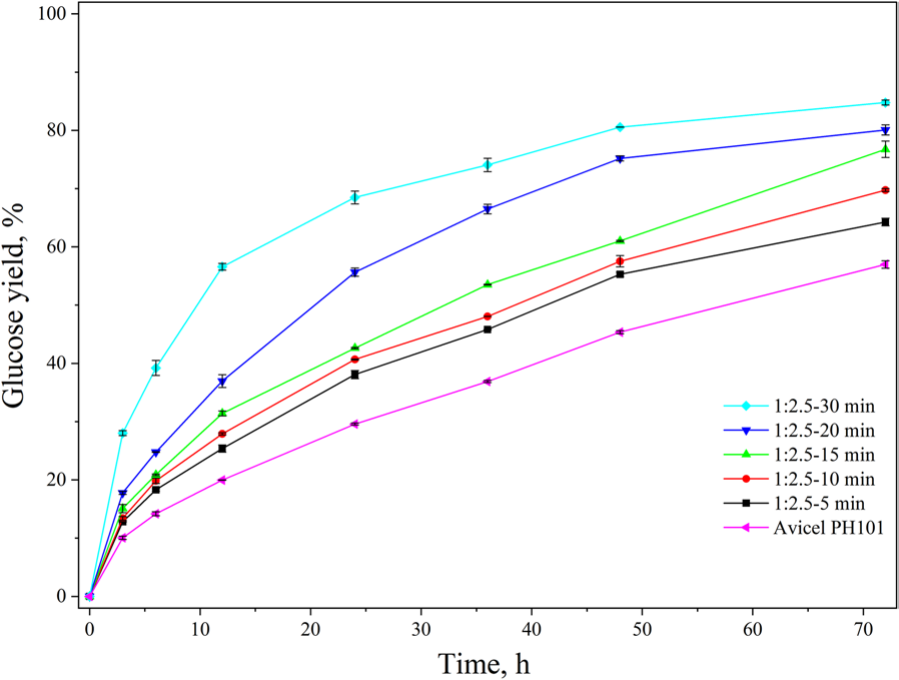
Enzymatic digestibility of cellulose after sulfuric acid treatment (cellulose-to-acid ratio of 1:2.5, hydrolysis condition: 2% solids loading, cellulase enzyme loading of 5 FPU/g cellulose, 150 rpm at 50 °C)

The glucan digestibility of sulfuric acid-treated Avicel was investigated in enzymatic hydrolysis tests under the following conditions: 2% solids loading, cellulase loading of 5 FPU/g cellulose, 150 rpm at 50 °C. The enzyme loading was much lower than the normal level of 15–20 FPU/g-glucan used in most biomass conversion studies [48, 49]. The results of the enzymatic hydrolysis of 1:2.5 acid-loading samples are shown in Fig. 7. For the raw Avicel, the glucose yield after 72 h of enzymatic hydrolysis was 56.98%. After 30 min of sulfuric acid treatment, the glucose yield increased to 84.78%. Not only the final glucose yield was increased, but also the rate of enzymatic hydrolysis accelerated significantly, especially at the early stage of hydrolysis. It took only 12 h for sample 1:2.5–30 min to achieve a yield of 56%, while it took 72 h for the raw Avicel to achieve the same level of yield. As shown in Fig. 7, the glucose yield increased with the increase in acid treatment time. On the other hand, it was found that the CrI increased as the acid treatment time was prolonged in this study (Fig. 5). The trend of glucose yield and CrI change found in this work was not in agreement with previous studies. A lot of researchers found that glucose yield has a strong negative correlation with the CrI of cellulose, i.e., lower CrI brings in higher glucose yields [29, 50]. We speculated that for cellulose treated with concentrated sulfuric acid, the increase of crystalline cellulose II content during cellulose regeneration in water after acid treatment increased CrI. Thus, the correlation between glucose yield and CrI in this study was the correlation between glucose yield and cellulose II content. Therefore, the most significant parameters affecting glucan digestibility were the cellulose II content and the DP for sulfuric acid-treated cellulose. The correlation between the glucose yield and the physicochemical characteristics of sulfuric acid-treated cellulose was analyzed, and the results of Pearson correlation analysis are shown in Table 3. The enzymatic glucose yield at 72 h was remarkably correlated with cellulose II content, DP, and CrI. The two close correlation coefficients of CrI and cellulose II content supported our speculation to some extent. As shown in Additional file 1: Fig. S5, the glucose yield had a strong linear relationship with CrI, cellulose II content, and DP. Their quantitative relationship was y (glucose yield) = 0.7185x (CrI) + 0.3784 (*R*^2^ = 0.7430), y (glucose yield) = 0.5034x (the content of cellulose II) + 0.3165 (*R*^2^ = 0.7026), and y (glucose yield) = − 0.0036x (DP) + 1.0293 (*R*^2^ = 0.7386), respectively. The enzymatic hydrolysis of samples of 1:2 and 1:3 acid loadings was very similar (see Additional file 1: Fig. S4).

**Table 3:** Pearson correlation analysis

	Cellulose-to-acid ratio	Treatment time	CrI	Content of cellulose II	D_50_	DP	Glucose yield
Cellulose-to-acid ratio	1	0	− 0.135	− 0.135	− 0.049	0.157	0.190
Treatment time		1	0.825^a^	0.867^a^	− 0.915^b^	− 0.885^a^	0.907^a^
CrI			1	0.943^a^	− 0.924^a^	− 0.893^a^	0.862^a^
Content of cellulose II				1	− 0.924^a^	− 0.890^a^	0.838^a^
D_50_					1	0.754	− 0.896^b^
DP						1	− 0.859^b^
Glucose yield							1

^a^Indicates extremely significant correlation between parameters (p < 0.01)

^b^Indicates significant correlation between parameters (p < 0.05)

**Fig. 8:**
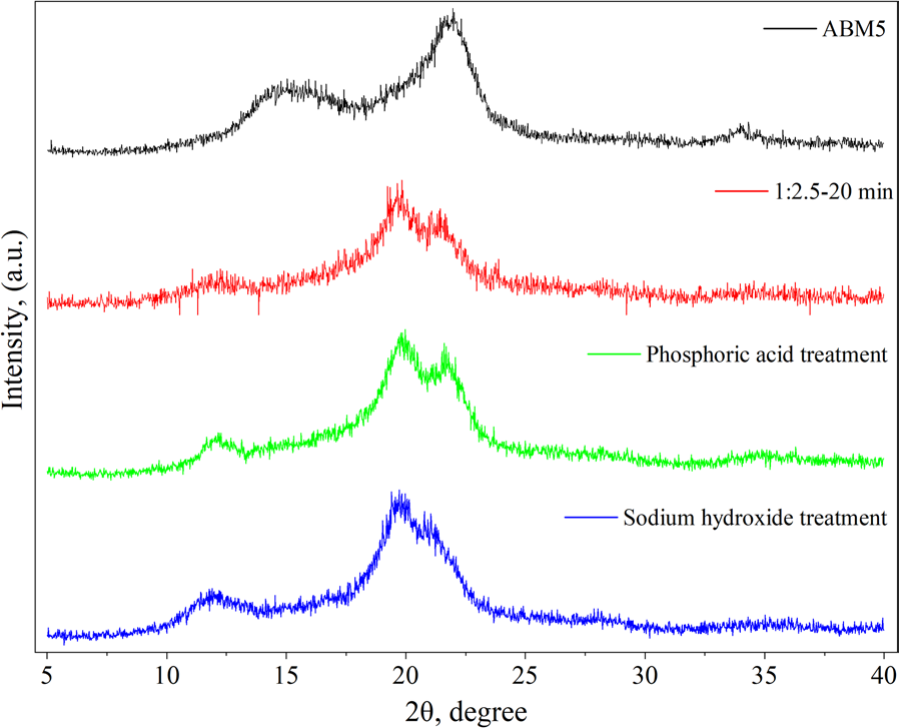
X-ray diffractograms of cellulose treated with phosphoric acid and sodium hydroxide, respectively

To further investigate the correlation between cellulose conversion and the cellulose II content as well as the CrI, cellulose was treated by four different methods: BM, sulfuric acid treatment, phosphoric acid treatment, and sodium hydroxide method to generate samples with similar CrI. The detailed methods for the latter two treatments were described by Wei et al. [37] and Isoga et al. [51], respectively. As shown in Fig. 8, Avicel after mechanical treatment of BM remained cellulose I structure, while all chemical treatments produced samples with cellulose II structure. Although all samples have similar CrI (Table 4), their behavior in enzymatic hydrolysis differed greatly. The glucan digestibility of the BM sample was 66.78%, and the sulfuric acid-treated sample gave a 79.55% glucose yield, which was 19.1% higher than the BM sample. The glucose yield for cellulose samples produced from phosphoric acid and sodium hydroxide was even higher at over 90%. Since these four samples had almost the same CrI, the significant difference in glucose yield could not be explained by CrI levels. This result was consistent with our speculation that the content of cellulose II was the major factor that influence the cellulose conversion.

**Table 4:** Comparison of glucose yield of different cellulose samples

Avicel treated by different methods	CrI, %	Cellulose II content, %	Glucose yield^a^, %
Ball milling	59.31 ± 1.01	0.00 ± 0.00	66.78 ± 0.35
Sulfuric acid	58.78 ± 2.14	96.55 ± 2.03	79.55 ± 0.87
Phosphoric acid	59.04 ± 1.96	100.00 ± 0.00	96.51 ± 0.73
Sodium hydroxide	58.52 ± 3.61	100.00 ± 0.00	91.97 ± 0.89

^a^Enzymatic hydrolysis condition: 2% solids loading, cellulase enzyme loading of 5 FPU/g cellulose, 150 rpm at 50 °C

## Conclusions

Low loadings of concentrated sulfuric acid were proven to be effective to break the recalcitrance of cellulose for enzymatic saccharification. The physicochemical properties of cellulose, such as DP, CrI, D_50_, surface morphology, and cellulose II content changed dramatically after 72% sulfuric acid treatment at room temperature. A high glucose yield of over 80% was achieved at a very low enzyme loading of 5 FPU/g-cellulose after acid treatment. The cellulose II content in cellulose was found to be closely related to its enzymatic saccharification yield. A positive correlation between cellulose CrI and glucose yield was found for concentrated sulfuric acid-treated cellulose, which was opposite to previous reports. The yield of glucose obtained by enzymatic hydrolysis of cellulose with the same crystallinity varies greatly with different treatments. The mechanism behind this phenomenon needs further investigation.

## Materials and methods

### Materials

Avicel PH101 (~ 50 μm particle size), which is microcrystalline cellulose, was purchased from Sigma Aldrich (Shanghai, China). Cellulase (Novozymes Cellic CTec2) was purchased from Sigma Aldrich (Shanghai, China). Sulfuric acid (H_2_SO_4_) AR grade was purchased from Sinopharm Group (Shanghai, China). All chemicals were used as received.

### Sulfuric acid treatment of cellulose

The treatment of cellulose with sulfuric acid was performed at room temperature. According to pre-experiments, sulfuric acid with a mass fraction of 72% was chosen to react with cellulose. Three different cellulose to H_2_SO_4_ loadings (1:2, 1:2.5, and 1:3) were investigated and the treatment time was up to 30 min. The mixing and reaction of cellulose and sulfuric acid were facilitated by a digital stirrer (IKA RW20, Germany) at 300 rpm. After being treated for a certain time, the cellulose–H_2_SO_4_ mixture was quickly transferred into 4 °C deionized water to stop the reaction. The water was kept stirring at 400 rpm to ensure even dispersion. The volume of water used in this step was five times the sulfuric acid volume. After stirring for 10 min, the dilute solution system was left at room temperature for 24 h before solid–liquid separation. After separation by centrifuge, the treated cellulose solid was washed with deionized water until its pH value reached near neutral. The solid was then freeze-dried to obtain the treated cellulose sample. The treated samples were named according to their treatment method, i.e. (cellulose to H_2_SO_4_ ratio)—(treatment time). For example, the sample 1:2.5–20 min refers to the sample treated with 2.5 times H_2_SO_4_ for 20 min. All acid treatment experiments were performed in duplicate.

### Ball milling of cellulose

The ball mill machine CJM-SY-B (Qinhuangdao Taiji Ring Nano Co., China) was used to prepare samples with varying degrees of crystallinity by mixing Avicel PH101 and zirconia balls (6–10 mm diameter) with a mass ratio of 1:30 for 5, 10, 15, 30, 60, and 120 min. The milling process is controlled below 20 °C using a cooling circulating water system. The samples obtained with different BM times are noted as ABM5, ABM10, ABM15, ABM30, ABM60, and ABM120, respectively. All ball-milling experiments were performed in duplicate.

### Enzymatic saccharification

The enzymatic hydrolysis of raw and treated cellulose was performed at 2% solid loading and a very low enzyme loading of 5 FPU/g cellulose. The detailed enzymatic hydrolysis process can be found in the standard operating procedure from NREL [52]. The supernatant after enzymatic digestion was used to determine the glucose concentration using an HPLC system (Waters, America) equipped with an RI detector by an Aminex HPX-87P column (Bio-Rad, USA). The mobile phase of ultrapure water was running at 0.6 mL/min. The enzymatic glucose yields were calculated as follows [52]:

$$\mathrm{Glucose\, yields }(\mathrm{\%})=\frac{\mathrm{c}\times \mathrm{V}\times 0.9}{\mathrm{m}}\times 100,$$

where c (mg/mL) is the concentration of glucose in the enzymatic digestion solution, V (mL) is the volume of the enzymatic digestion solution, and m (mg) is the weight of the cellulose samples added in the enzymatic digestion. All enzymatic saccharification experiments were performed in duplicate.

### XRD analysis of cellulose samples

XRD measurements were performed with an XD3 polycrystalline X-ray diffractometer (PERSEE, China) with Cu Kα radiation at 36 kV and 20 mA. The scanning range of 2θ was from 5 to 40° at a rate of 2°/min in 0.02° increments. Each sample was measured in duplicate. The CrI of the samples was calculated according to the method of Segal et al. and Azubuike et al. [53, 54] as shown below:

$$\mathrm{CrI }\left(\mathrm{\%}\right)=\frac{{I}_{002}-{I}_{am}}{{I}_{002}}\times 100,$$

where *I*_*002*_ is the maximum intensity of the lattice diffraction of the main peak (002) (at 22.4° for cellulose I and 21.7° for cellulose II), and *I*_*am*_ is the intensity of amorphous cellulose (at 18° for cellulose I and 16° for cellulose II, respectively), as shown in Additional file 1: Fig. S6.

The content of cellulose II in the samples was determined using the XRD calibration method of the inner standards [55]:

$$\mathrm{Content\, of\, cellulose II }\left(\mathrm{\%}\right)=200\times \frac{{I}_{12}}{{I}_{15}+{I}_{16}},$$

where *I*_*12*_, *I*_*15*_, and *I*_*16*_ are the diffraction intensities at the 2θ angle range 12, 15, and 16 degrees, respectively.

### FTIR analysis of cellulose samples

The preparation of the cellulose sample for the FTIR test was the same as the reference [35]. Each sample was measured in duplicate. The infrared crystallinity ratio of the samples was calculated according to the method of Nelson and O’Connor [43]. The calculation was as follows:

$$\mathrm{Infrared\, crystallinity \, ratio}=\frac{{A}_{{1372cm}^{-1}}}{{A}_{{2900cm}^{-1}}},$$

where ${A}_{{2900cm}^{-1}}$ and ${A}_{{1372cm}^{-1}}$ are the absorptivity of the 2900 cm^−1^ and 1372 cm^−1^ bands, respectively.

### Particle size measurement of cellulose samples

The particle size of cellulose was measured using MASTERSIZER 3000 (Malvern, UK). Each sample was repeated twice, with each repeat measured five times. The median particle diameter D_50_ was chosen to represent the particle size distribution. D_50_ is the particle size that corresponds to the cumulative percentage of 50%.

### DP measurement of cellulose samples

The DP was determined by calculating the ratio of glucose monomer concentration to the reducing end concentration. The glucose monomer concentration was determined using the phenol–sulfuric acid method, and the molar concentration of the reduced end of cellulose was determined by the modified BCA method [25]. Each sample was measured in duplicate.

### Scanning electron microscopy (SEM) analysis of cellulose samples

The surface morphological characteristics of the cellulose were observed with Hitachi SU3500 (Hitachi, Japan). The samples were evenly adhered to the carbon tape and gold sprayed for 2 min before SEM observation.

## Supplementary Information

**Additional file 1: Fig. S1.** SEM images of cellulose samples (a) Avicel PH101, (b) 1:2-5 min, (c) 1:2 -30 min, (d) 1:3-5 min, (e) 1:3-30 min. **Fig. S2.** X-ray diffractograms of sulfuric acid-treated cellulose samples (cellulose-to-acid ratio of 1:2 and 1:3). **Fig. S3.** FTIR spectra of cellulose samples after sulfuric acid treatment (cellulose-to-acid ratio of 1:2 and 1:3). **Fig. S4.** Enzymatic digestibility of cellulose after sulfuric acid treatment, cellulose-to-acid ratio of 1:2 (left) and 1:3 (right), hydrolysis condition: 2% solids loading, cellulase enzyme loading of 5 FPU/g cellulose, 150 rpm at 50 °C. **Fig. S5.** Relationship of glucose yield with CrI (left), content of cellulose II (middle), and DP (right). **Fig. S6**. Illustrative demonstration of the peak heights of cellulose I and cellulose II.

## Data Availability

All data generated or analyzed during this study are included in this published article.

## References

[1] Carere CR (2008). Third generation biofuels via direct cellulose fermentation. Int J Mol Sci.

[2] Lynd LR (2017). The grand challenge of cellulosic biofuels. Nat Biotechnol.

[3] Artz J, Palkovits R (2018). Cellulose-based platform chemical: the path to application. Curr Opin Green Sustain Chem.

[4] Wang S, Lu A, Zhang L (2016). Recent advances in regenerated cellulose materials. Prog Polym Sci.

[5] Srivastava RK (2021). Biomass utilization and production of biofuels from carbon neutral materials. Environ Pollut.

[6] Mathews JA (2008). Carbon-negative biofuels. Energy Policy.

[7] Efroymson RA. et al. Billion-ton report: advancing domestic resources for a thriving bioeconomy, volume 2: environmental sustainability effects of select scenarios from volume 1*.* 2017.

[8] Klemm D (2005). Cellulose: fascinating biopolymer and sustainable raw material. Angew Chem Int Ed.

[9] Yue Y (2015). Characterization of cellulose I/II hybrid fibers isolated from energycane bagasse during the delignification process: morphology, crystallinity and percentage estimation. Carbohydr Polym.

[10] Kabir SF, Naeem M, Aftab T, Masroor M, Khan A (2022). Occurrence, distribution, and structure of natural polysaccharides. Radiation-processed polysaccharides.

[11] Gong J (2017). Research on cellulose nanocrystals produced from cellulose sources with various polymorphs. RSC Adv.

[12] Zugenmaier P (2001). Conformation and packing of various crystalline cellulose fibers. Prog Polym Sci.

[13] Hayashi J (1975). The confirmation of existences of cellulose IIII, IIIII, IVI, and IVII by the X-ray method. J Polym Sci: Polym Lett Ed.

[14] Gardiner ES, Sarko A (1985). Packing analysis of carbohydrates and polysaccharides. 16. The crystal structures of celluloses IVI and IVII. Can J Chem.

[15] Astruc J (2021). Rod- and sphere-shaped cellulose nanocrystals (CNCs) type-II derived from Asclepias syriaca stem residues: composition, morphology, and thermal properties. Can J Chem.

[16] Moxley G, Zhu Z, Zhang YHP (2008). Efficient sugar release by the cellulose solvent-based lignocellulose fractionation technology and enzymatic cellulose hydrolysis. J Agric Food Chem.

[17] Janga KK, Hagg MB, Moe S (2012). Influence of acid concentration, temperature, and time on decrystallization in two-stage concentrated sulfuric acid hydrolysis of pinewood and aspenwood: a statistical approach. BioResources.

[18] Macrae R, Robinson RK, Sadler MJ (1993). Encyclopaedia of food science, food technology and nutrition.

[19] Saeman JF, Bubl JL, Harris EE (1945). Quantitative saccharification of wood and cellulose. Ind Eng Chem Anal Ed.

[20] Lee YY. Use of non-crystalline cellulose as a medicine tablet medium. Google Patents. 2011.

[21] Dong XM, Revol J-F, Gray DG (1998). Effect of microcrystallite preparation conditions on the formation of colloid crystals of cellulose. Cellulose.

[22] Sluiter JB (2010). Compositional analysis of lignocellulosic feedstocks. 1. Review and description of methods. J Agric Food Chem.

[23] Yeh A-I, Huang Y-C, Chen SH (2010). Effect of particle size on the rate of enzymatic hydrolysis of cellulose. Carbohyd Polym.

[24] Wiman M (2012). Cellulose accessibility determines the rate of enzymatic hydrolysis of steam-pretreated spruce. Biores Technol.

[25] Zhang YHP, Lynd LR (2005). Determination of the number-average degree of polymerization of cellodextrins and cellulose with application to enzymatic hydrolysis. Biomacromol.

[26] Jiang W (2016). Changes on structural properties of biomass pretreated by combined deacetylation with liquid hot water and its effect on enzymatic hydrolysis. Bioresour Technol.

[27] Peng H (2013). A novel combined pretreatment of ball milling and microwave irradiation for enhancing enzymatic hydrolysis of microcrystalline cellulose. Biores Technol.

[28] Ioelovich M, Morag E (2011). Effect Of cellulose structure on enzymatic hydrolysis. BioResources.

[29] Kuo C-H, Lee C-K (2009). Enhancement of enzymatic saccharification of cellulose by cellulose dissolution pretreatments. Carbohyd Polym.

[30] Liu Z (2015). Preparation and characterization of regenerated cellulose from ionic liquid using different methods. Carbohyd Polym.

[31] Ji G (2017). Quantitative approaches for illustrating correlations among the mechanical fragmentation scales, crystallinity and enzymatic hydrolysis glucose yield of rice straw. Biores Technol.

[32] Park S (2010). Cellulose crystallinity index: measurement techniques and their impact on interpreting cellulase performance. Biotechnol Biofuels.

[33] Nam S (2016). Segal crystallinity index revisited by the simulation of X-ray diffraction patterns of cotton cellulose Iβ and cellulose II. Carbohyd Polym.

[34] Marco M (2018). Effect of ball-milling on crystallinity index, degree of polymerization and thermal stability of cellulose. Bioresour Technol.

[35] Yang J (2022). Effect of combined wet alkaline mechanical pretreatment on enzymatic hydrolysis of corn stover and its mechanism. Biotechnol Biofuels Bioprod.

[36] Dawsey TR (1994). Cellulosic polymers, blends and composites.

[37] Wei S, Kumar V, Banker GS (1996). Phosphoric acid mediated depolymerization and decrystallization of cellulose: preparation of low crystallinity cellulose —A new pharmaceutical excipient. Int J Pharm.

[38] Ouyang P (2009). Dissolution of microcrystalline cellulose in phosphoric acid—Molecular changes and kinetics. Molecules.

[39] Hashaikeh R, Abushammala H (2011). Acid mediated networked cellulose: preparation and characterization. Carbohyd Polym.

[40] Alemdar A, Sain M (2008). Isolation and characterization of nanofibers from agricultural residues—Wheat straw and soy hulls. Bioresour Technol.

[41] Kaushik A, Singh M (2011). Isolation and characterization of cellulose nanofibrils from wheat straw using steam explosion coupled with high shear homogenization. Carbohydr Res.

[42] Sun XF (2005). Characteristics of degraded cellulose obtained from steam-exploded wheat straw. Carbohyd Res.

[43] Nelson ML, O’Connor RT (1964). Relation of certain infrared bands to cellulose crystallinity and crystal lattice type. Part II. A new infrared ratio for estimation of crystallinity in celluloses I and II. J Appl Polym Sci.

[44] Zhang L, Ruan D, Zhou J (2001). Structure and properties of regenerated cellulose films prepared from cotton linters in NaOH/Urea aqueous solution. Ind Eng Chem Res.

[45] Ruan D (2004). Microporous membranes prepared from cellulose in NaOH/thiourea aqueous solution. J Membr Sci.

[46] Han J (2013). Self-assembling behavior of cellulose nanoparticles during freeze-drying: effect of suspension concentration, particle size, crystal structure, and surface charge. Biomacromol.

[47] Stubičar N (1998). An X-ray diffraction study of the crystalline to amorphous phase change in cellulose during high-energy dry ball milling. Holzforschung.

[48] Baral P (2020). Expeditious production of concentrated glucose-rich hydrolysate from sugarcane bagasse and its fermentation to lactic acid with high productivity. Food Bioprod Process.

[49] Yadav N, Nain L, Khare SK (2021). One-pot production of lactic acid from rice straw pretreated with ionic liquid. Biores Technol.

[50] Tyufekchiev M (2019). Reaction engineering implications of cellulose crystallinity and water-promoted recrystallization. Green Chem.

[51] Isogai A, Atalla RH (1998). Dissolution of cellulose in aqueous NaOH solutions. Cellulose.

[52] Resch MG, Baker JO, Decker SR (2015). Enzymatic saccharification of lignocellulosic biomass.

[53] Azubuike CP (2011). Physicochemical properties of maize cob cellulose powders reconstituted from ionic liquid solution. Cellulose.

[54] Segal L, Creely JJ, Martin AE, Conrad CM (1959). An empirical method for estimating the degree of crystallinity of native cellulose using the X-ray diffractometer. Text Res J.

[55] Ioelovich MY, Veveris GP (1983). Cellulose II content determination by X-ray analysis using an internal standard. Wood Chem.

