# Supplementary material for: The transformations of cellulose after concentrated sulfuric acid treatment and its impact on the enzymatic saccharification
Source: Biotechnol Biofuels Bioprod. 2023 Mar 4;16:36. doi: 10.1186/s13068-023-02293-4 (PMC9985267; doi:10.1186/s13068-023-02293-4)
Supplement: Supplementary file 1 — Additional file 1: Fig. S1. SEM images of cellulose samples (a) Avicel PH101, (b) 1:2-5 min, (c) 1:2 -30 min, (d) 1:3-5 min, (e) 1:3-30 min. Fig. S2. X-ray diffractograms of sulfuric acid-treated cellulose samples (cellulose-to-acid ratio of 1:2 and 1:3). Fig. S3. FTIR spectra of cellulose samples after sulfuric acid treatment (cellulose-to-acid ratio of 1:2 and 1:3). Fig. S4. Enzymatic digestibility of cellulose after sulfuric acid treatment, cellulose-to-acid ratio of 1:2 (left) and 1:3 (right), hydrolysis condition: 2% solids loading, cellulase enzyme loading of 5 FPU/g cellulose, 150 rpm at 50 °C. Fig. S5. Relationship of glucose yield with CrI (left), content of cellulose II (middle), and DP (right). Fig. S6. Illustrative demonstration of the peak heights of cellulose I and cellulose II. [file 13068_2023_2293_MOESM1_ESM.docx]

**Additional file**


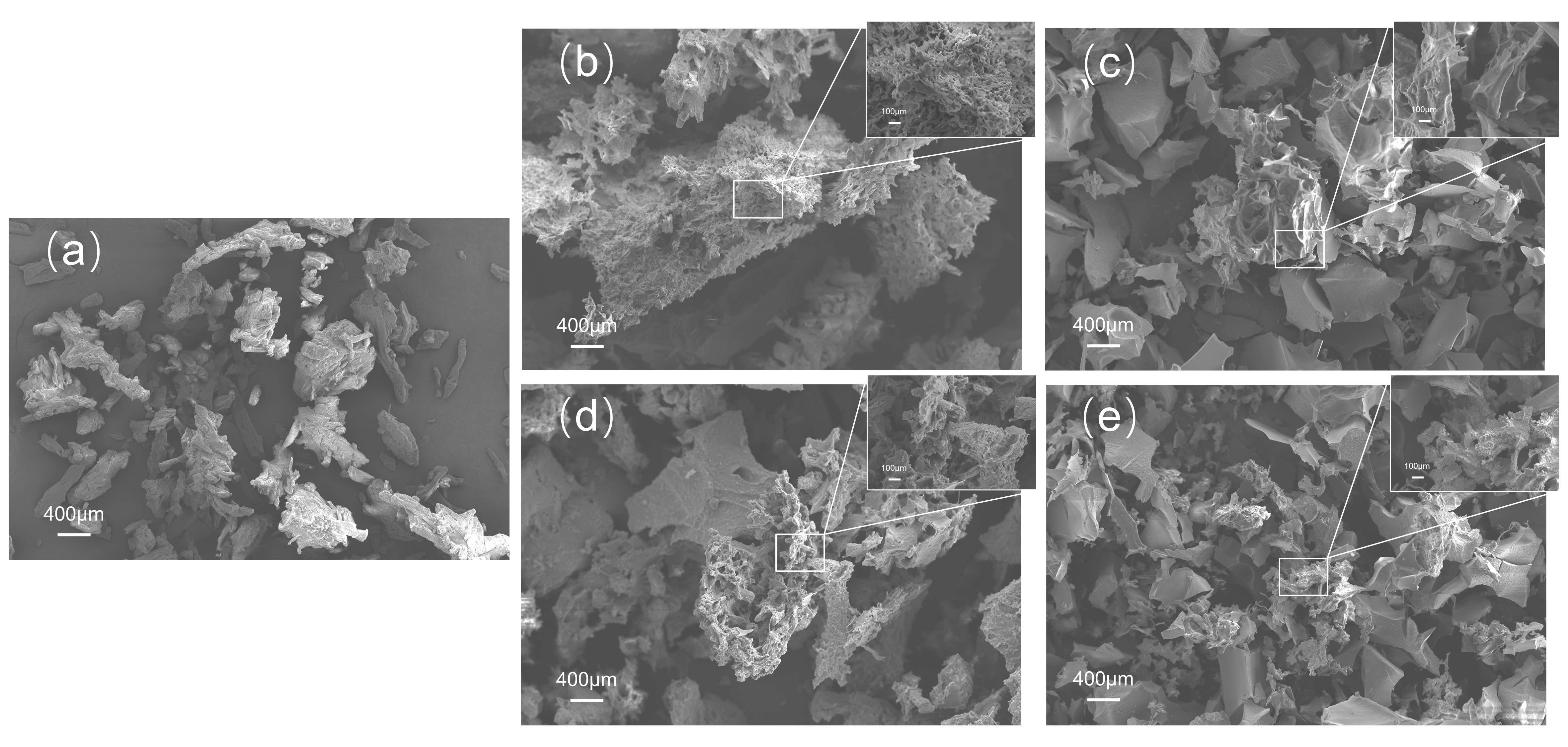


Fig. S1 SEM images of cellulose samples (a) Avicel PH101, (b) 1:2-5 min, (c) 1:2 -30 min, (d) 1:3-5 min, (e) 1:3-30 min.


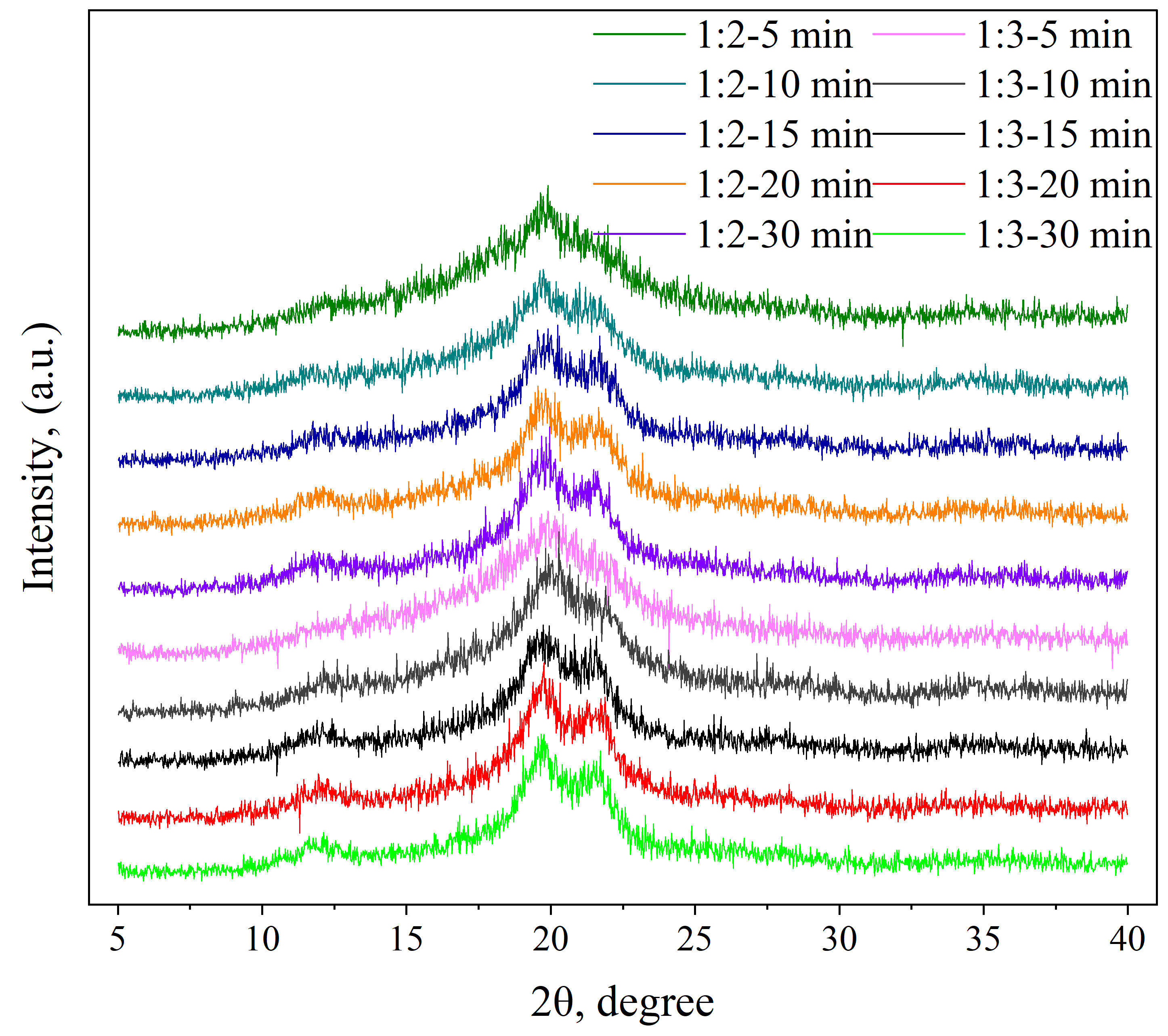


Fig. S2 X-ray diffractograms of sulfuric acid-treated cellulose samples (cellulose-to-acid ratio of 1:2 and 1:3).


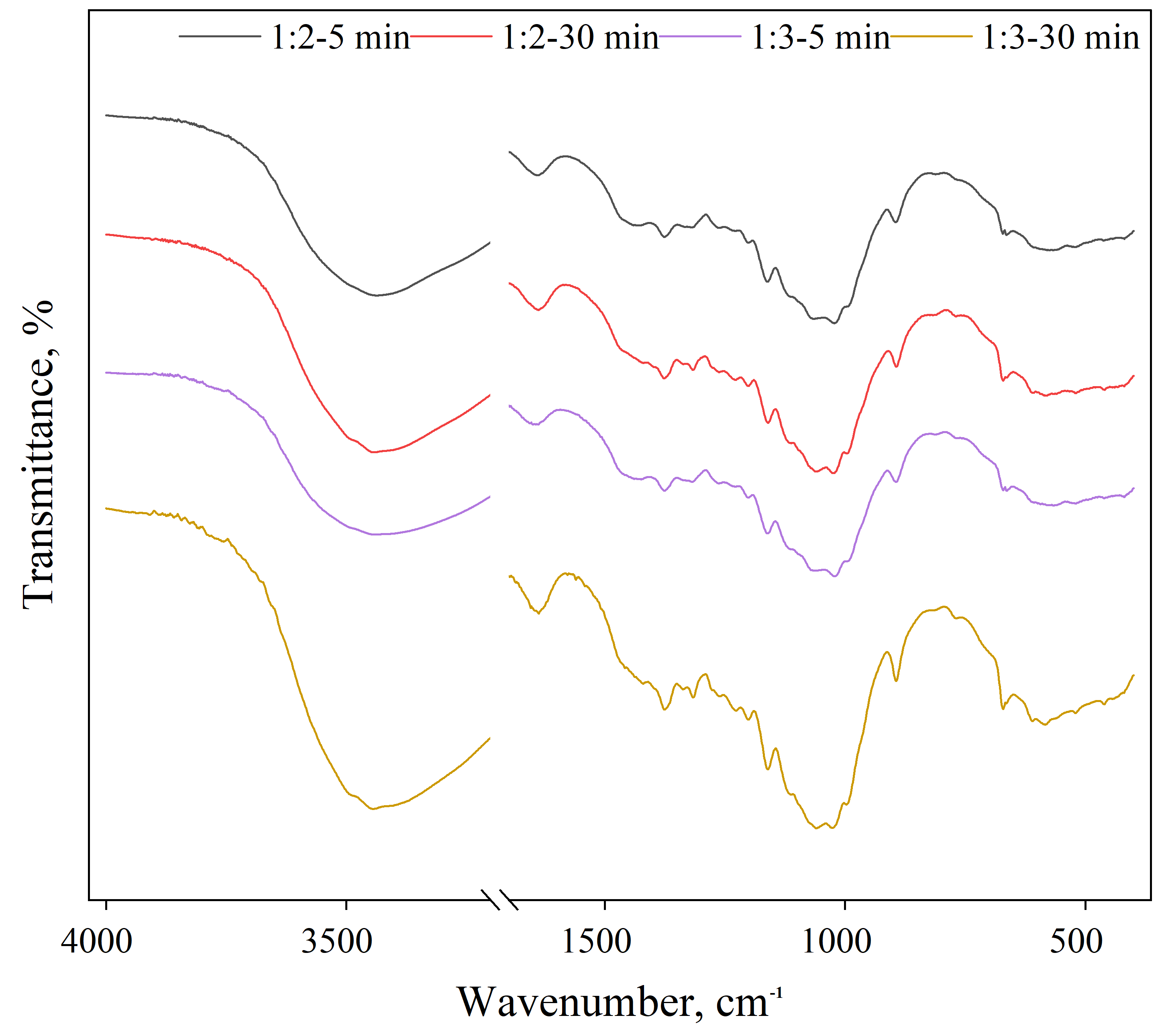


Fig. S3 FTIR spectra of cellulose samples after sulfuric acid treatment (cellulose-to-acid ratio of 1:2 and 1:3).


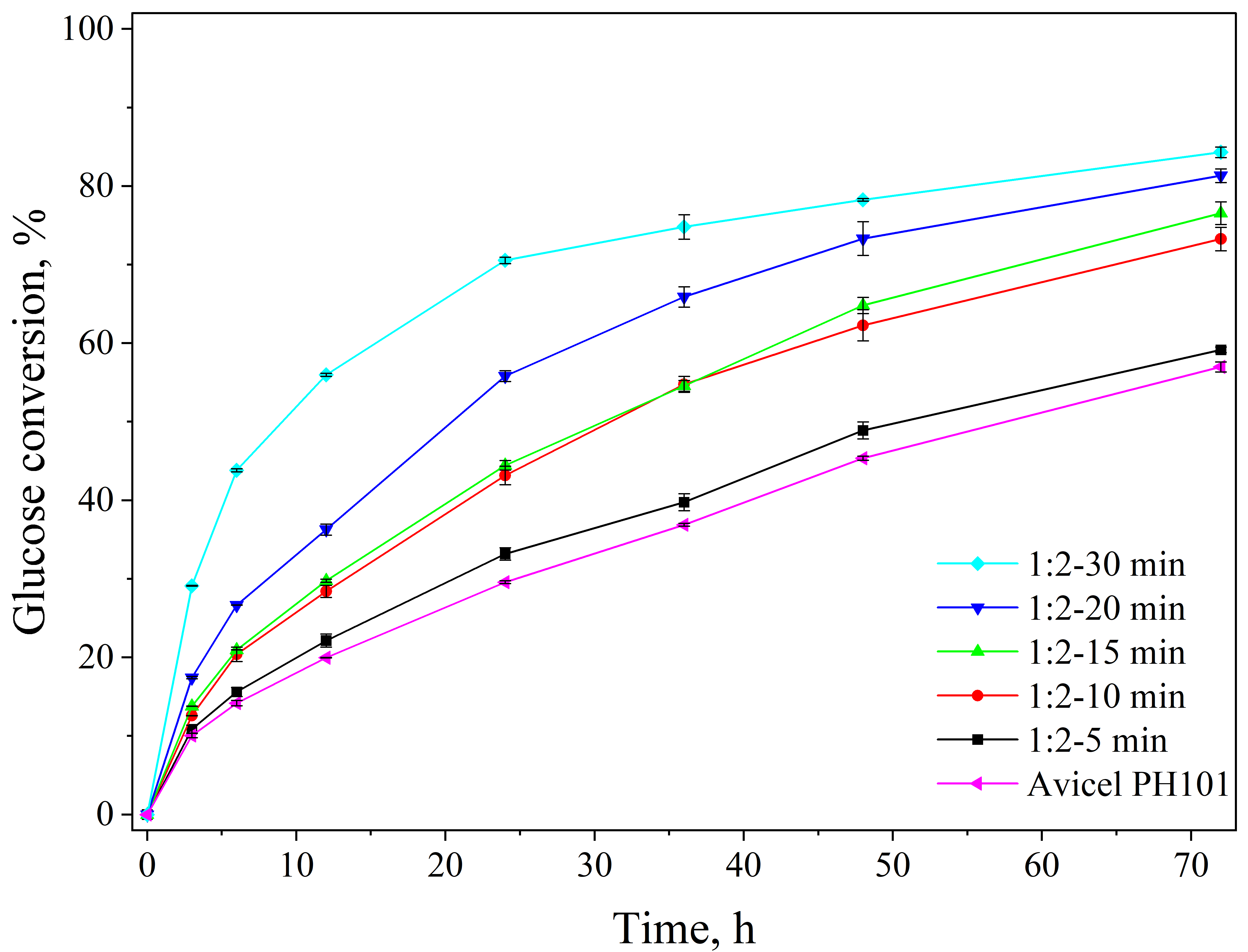

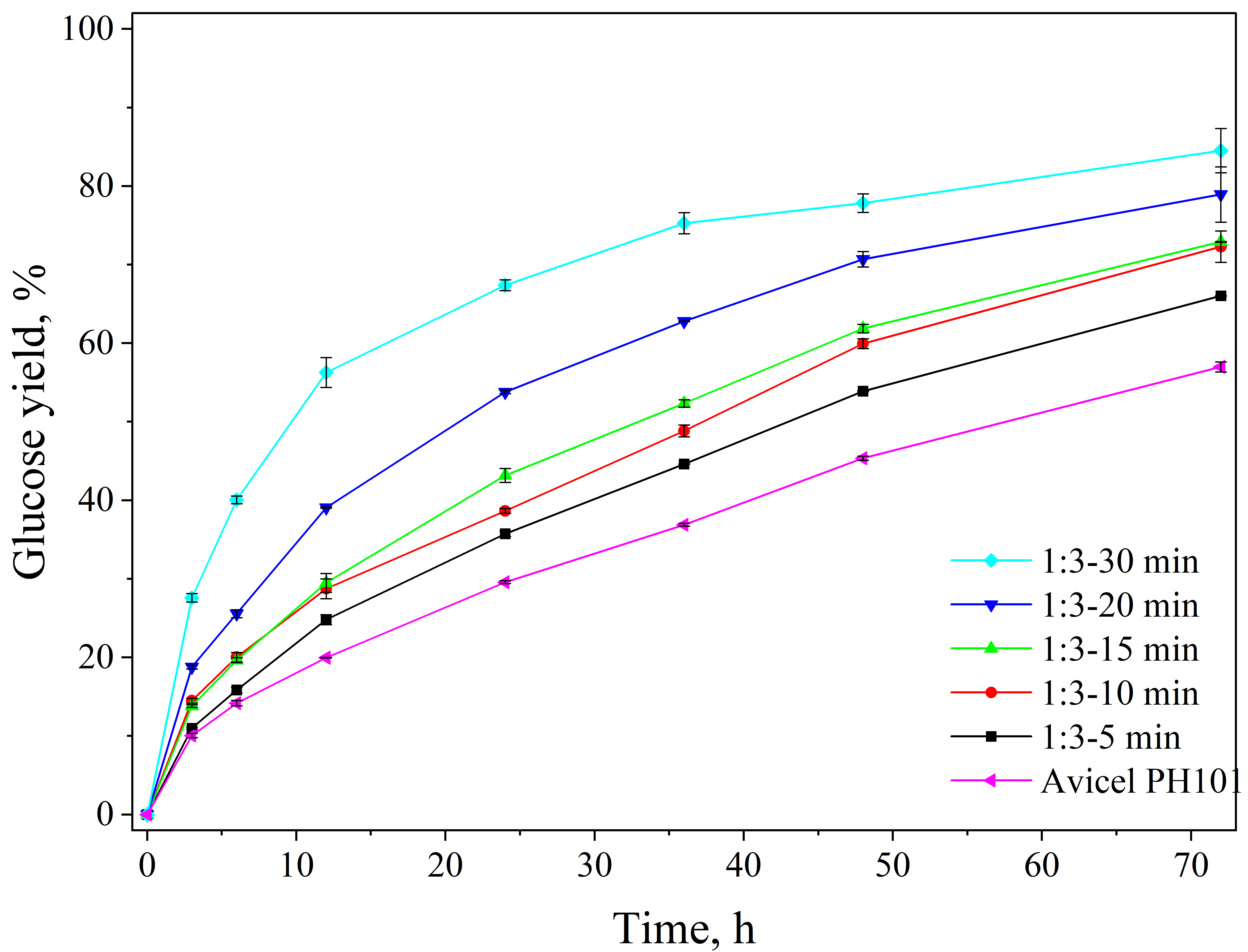


Fig. S4 Enzymatic digestibility of cellulose after sulfuric acid treatment, cellulose-to-acid ratio of 1:2 (left) and 1:3 (right), hydrolysis condition: 2% solids loading, cellulase enzyme loading of 5 FPU/g cellulose, 150 rpm at 50 °C.


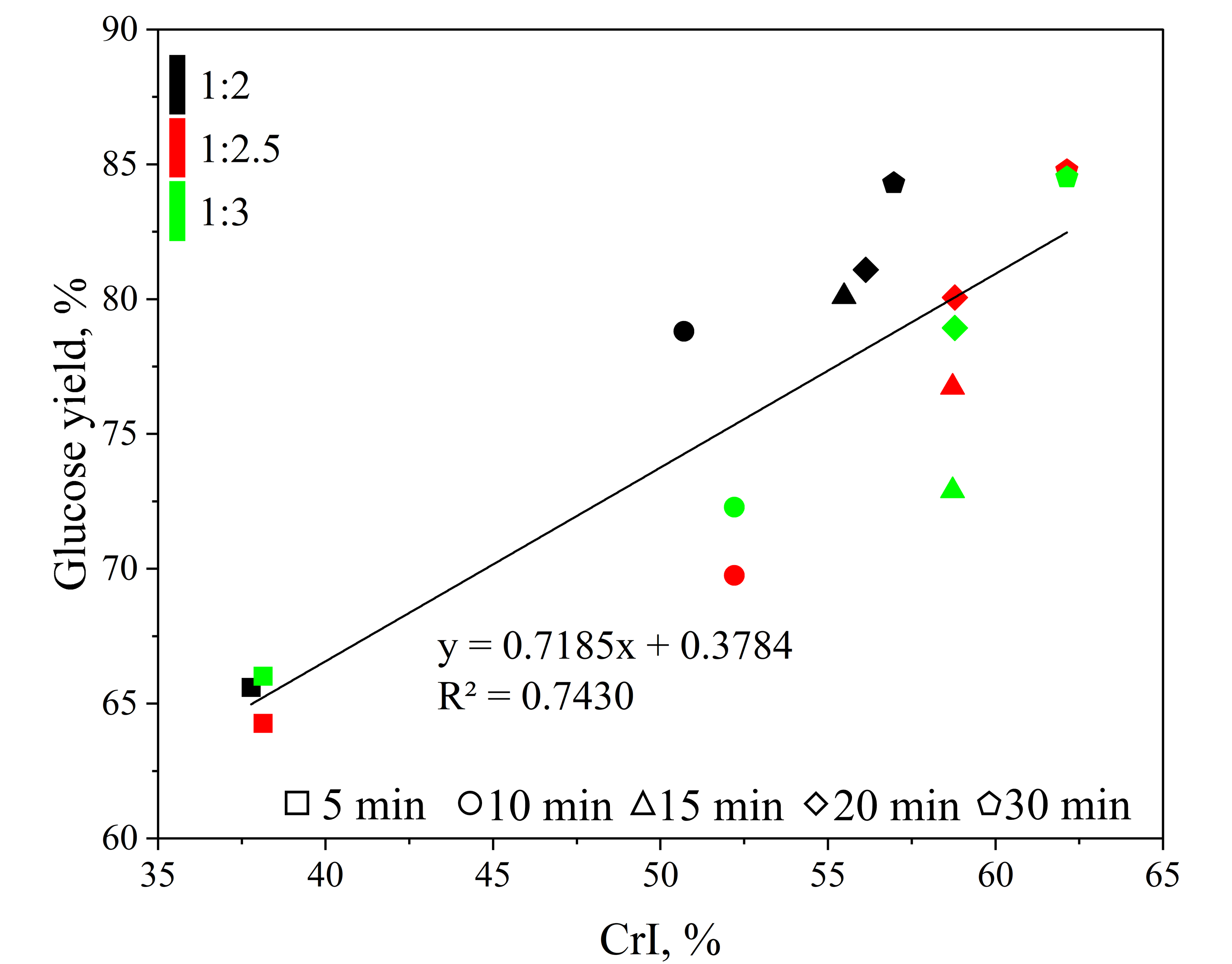

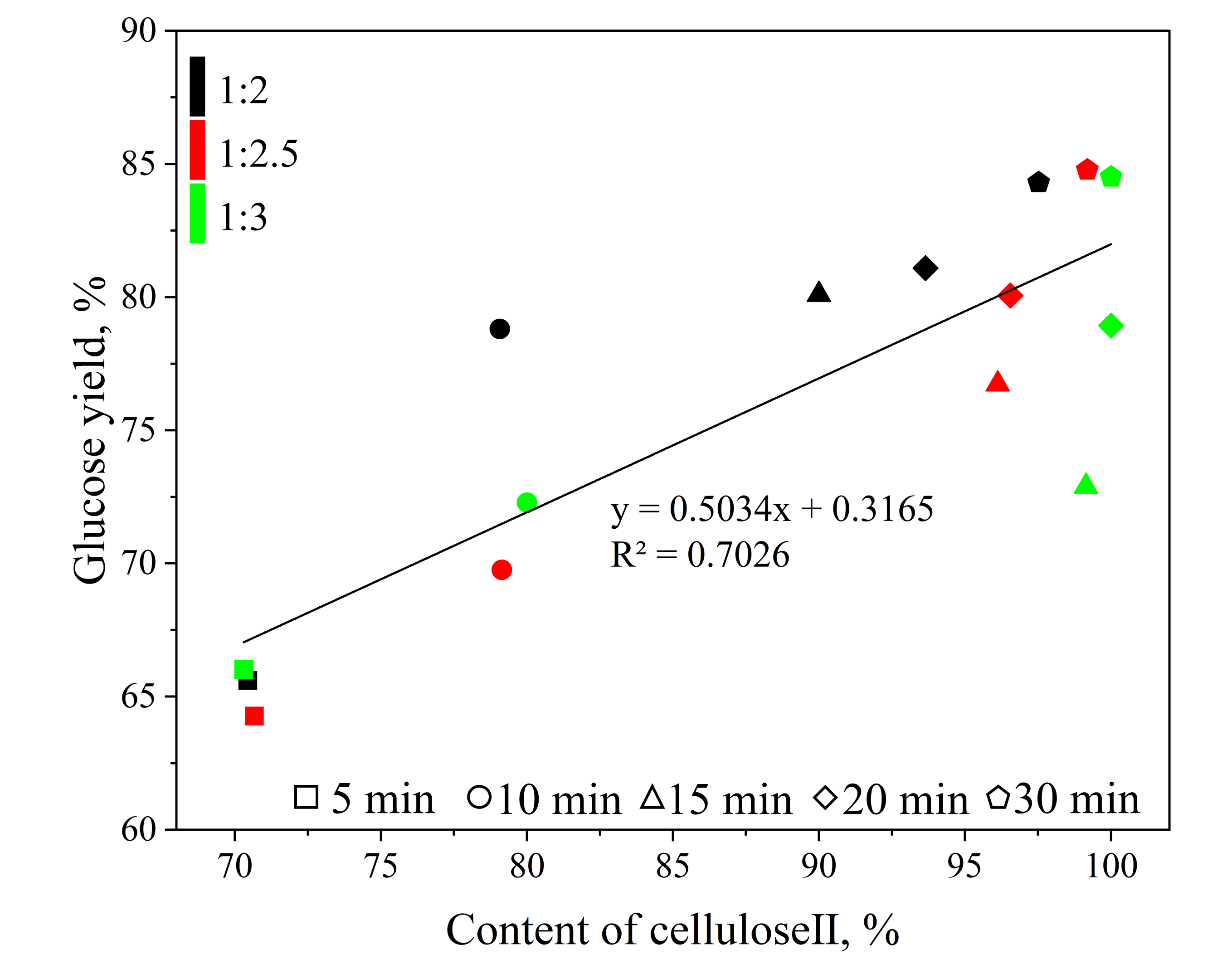

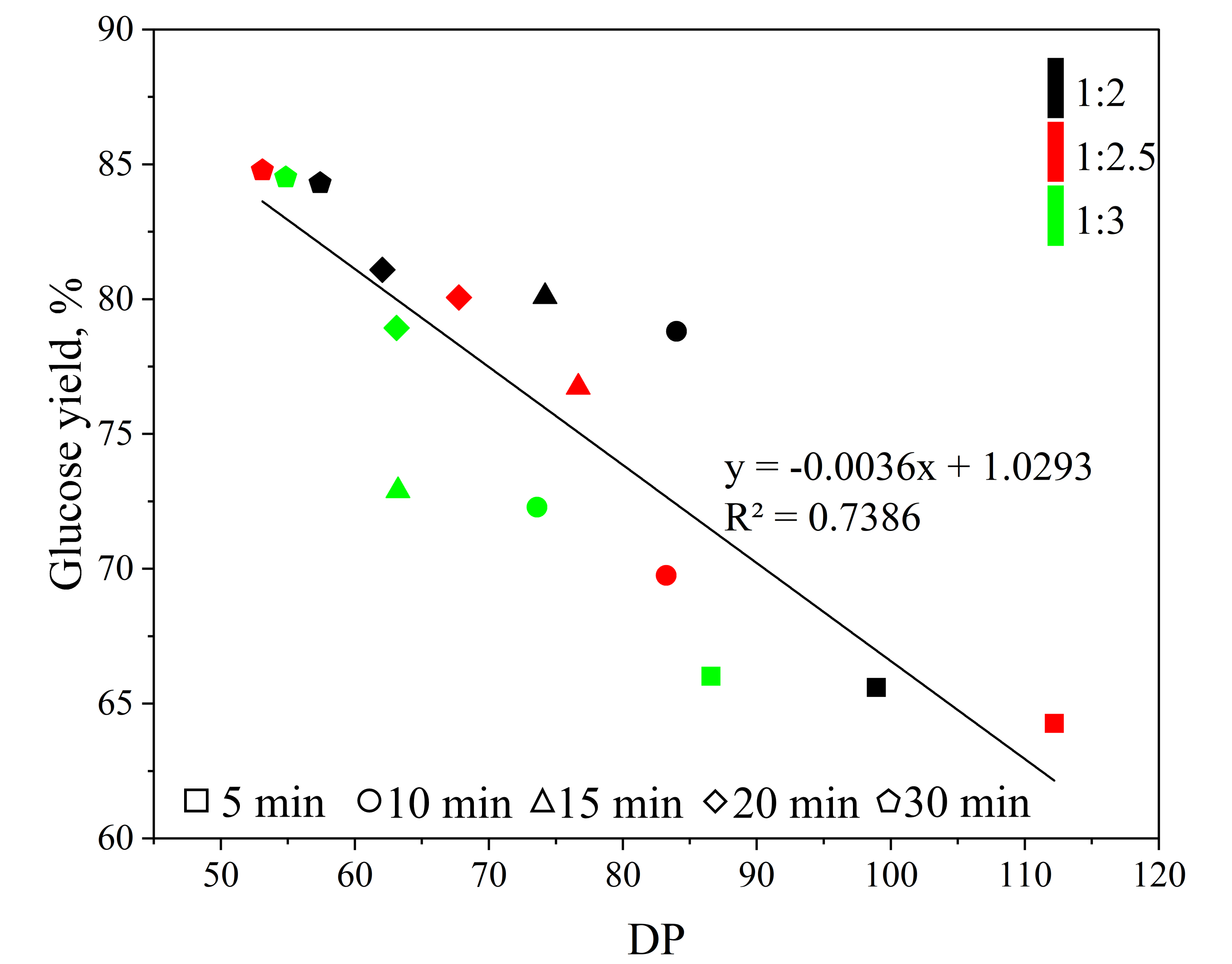


Fig. S5 Relationship of glucose yield with CrI (left), content of cellulose Ⅱ (middle), and DP (right).


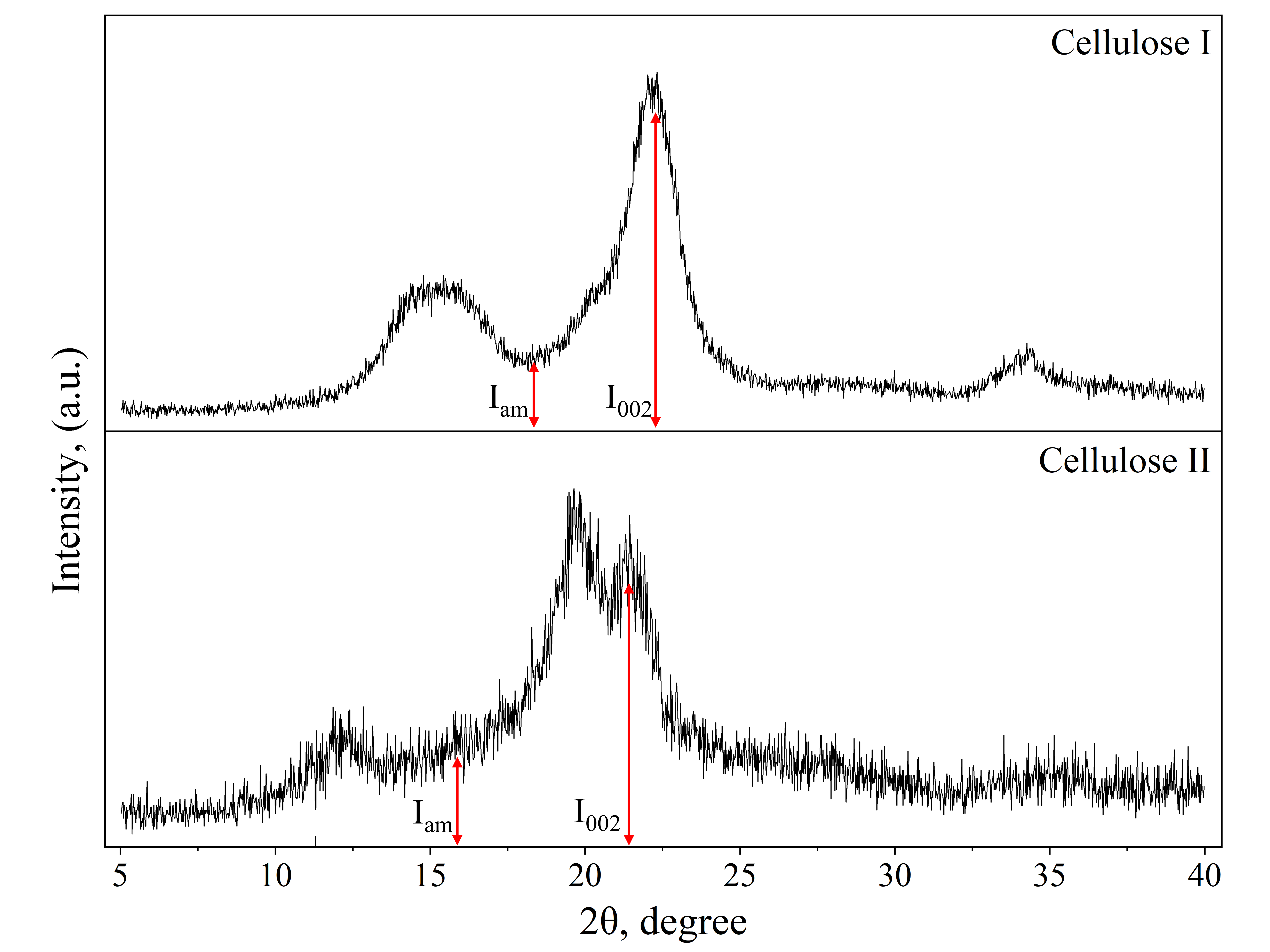


Fig. S6 Illustrative demonstration of the peak heights of cellulose I and cellulose II.
